# DiaFit: The Development of a Smart App for Patients with Type 2 Diabetes and Obesity

**DOI:** 10.2196/diabetes.6662

**Published:** 2016-12-13

**Authors:** François Modave, Jiang Bian, Eric Rosenberg, Tonatiuh Mendoza, Zhan Liang, Ravi Bhosale, Carlos Maeztu, Camila Rodriguez, Michelle I Cardel

**Affiliations:** 1 Department of Health Outcomes and Policy University of Florida Gainesville, FL United States; 2 Department of Internal Medicine University of Florida Gainesville, FL United States

**Keywords:** mHealth, diabetes, obesity, apps

## Abstract

**Background:**

Optimal management of chronic diseases, such as type 2 diabetes (T2D) and obesity, requires patient-provider communication and proactive self-management from the patient. Mobile apps could be an effective strategy for improving patient-provider communication and provide resources for self-management to patients themselves.

**Objective:**

The objective of this paper is to describe the development of a mobile tool for patients with T2D and obesity that utilizes an integrative approach to facilitate patient-centered app development, with patient and physician interfaces. Our implementation strategy focused on the building of a multidisciplinary team to create a user-friendly and evidence-based app, to be used by patients in a home setting or at the point-of-care.

**Methods:**

We present the iterative design, development, and testing of DiaFit, an app designed to improve the self-management of T2D and obesity, using an adapted Agile approach to software implementation. The production team consisted of experts in mobile health, nutrition sciences, and obesity; software engineers; and clinicians. Additionally, the team included citizen scientists and clinicians who acted as the de facto software clients for DiaFit and therefore interacted with the production team throughout the entire app creation, from design to testing.

**Results:**

DiaFit (version 1.0) is an open-source, inclusive iOS app that incorporates nutrition data, physical activity data, and medication and glucose values, as well as patient-reported outcomes. DiaFit supports the uploading of data from sensory devices via Bluetooth for physical activity (iOS step counts, FitBit, Apple watch) and glucose monitoring (iHealth glucose meter). The app provides summary statistics and graphics for step counts, dietary information, and glucose values that can be used by patients and their providers to make informed health decisions. The DiaFit iOS app was developed in Swift (version 2.2) with a Web back-end deployed on the Health Insurance Portability and Accountability Act compliant-ready Amazon Web Services cloud computing platform. DiaFit is publicly available on GitHub to the diabetes community at large, under the GNU General Public License agreement.

**Conclusions:**

Given the proliferation of health-related apps available to health consumers, it is essential to ensure that apps are evidence-based and user-oriented, with specific health conditions in mind. To this end, we have used a software development approach focusing on community and clinical engagement to create DiaFit, an app that assists patients with T2D and obesity to better manage their health through active communication with their providers and proactive self-management of their diseases.

## Introduction

Between 1980 and 2014, the number of Americans diagnosed with diabetes increased fourfold [1]. Almost 90% of individuals with type 2 diabetes (T2D) are obese, and the global epidemic of T2D is largely explained by the dramatic increase in both the incidence and prevalence of obesity over the past 40 years. The excess lifetime medical spending for individuals with T2D is up to US $211,400 [2], and aggregate obesity-related medical care costs in the United States reached a staggering US $147 billion in 2008.

Poor nutrition, low levels of physical activity, and sedentary lifestyles contribute greatly to T2D and obesity [3-10]. Current national estimates show that close to 64% Americans are trying to lose weight, and that nearly half are actively engaged in a weight loss program [11]. However, primary care providers are traditionally not trained to provide expertise pertaining to physical activity and nutrition, which could aid in weight loss and improved glucose control. Therefore, it is essential to develop comprehensive novel approaches that complement clinical care, to help patients with T2D and obesity manage their conditions, and to reduce long-term T2D and obesity complications. Longer-term lifestyle interventions and behavioral modifications have been found to reduce body weight and T2D complications, including: self-monitoring of weight, dietary intake, activity, and blood glucose; and medication compliance [12]. However, these interventions are often costly and resource intensive, and lack sustainability components. Management of T2D and obesity is self-directed, as individuals need to make day-to-day decisions related to controlling their chronic diseases [13]. Cost-effective and sustainable interventions to improve T2D and obesity-related outcomes could be achieved at a relatively low cost [14], and could save hundreds of thousands of dollars at the individual level and hundreds of billions of dollars at a national level [2].

The ubiquitous nature of the Internet and mobile technologies makes them potential cost-effective and sustainable tools to improve health knowledge and outcomes for chronic diseases, such as T2D and obesity. According to the PEW Research Center, 70% of Americans have access to high-speed Internet at home [1] and 64% have access to a smartphone [15]. Growing evidence indicates that digital media (apps in particular) have the potential to be effective and scalable approaches to deliver health behavior interventions across the socioeconomic gradient [2,3,14,16-18]. However, research assessing the quality of health-related apps suggests that many of these apps lack the evidence-based standards necessary in health care [4-9]. A possible explanation for this lack of standards is that most apps are not necessarily developed with the end-user in mind, and their implementation is undertaken without patient or expert (eg, physician) input.

The primary goal of this paper is to present DiaFit, a T2D and obesity-focused iOS app, and its implementation process, involving its potential end users. The app was developed to help patients self-manage T2D and obesity, and allow physicians to keep track of their patients’ progress. The majority of iOS apps targeting diabetes lack evidence-based support, functionalities, and interfacing with devices that support standard wireless communication protocols (eg, Bluetooth, Bluetooth Low Energy [BLE], or ANT+) [6,10,11]. This limitation forces patients with T2D and obesity to use multiple apps to address the various aspects of their chronic conditions. This scenario is far from ideal, since each app is designed differently and typically comes with a learning curve before the app can be used adequately. Moreover, dealing with multiple apps might prevent the user from understanding the interactions between nutrition, medication, and glucose levels. Therefore, we developed DiaFit, an app that allows a user to store their dietary intake, physical activity log, medication use, blood glucose values, and general well-being in one app. DiaFit permits seamless uploads via Bluetooth when possible. Finally, there is evidence that when building such mobile apps, patients and physicians should be involved, in particular for health-related apps that are aimed at older adults [10]. Therefore, DiaFit was developed in close collaboration with key stakeholders, including a primary care physician, citizen scientists, and people with diabetes and/or obesity; all of whom acted as potential users. Citizen scientists are defined as lay people who engage in scientific research . In our project these individuals were paid for their time and their contributions were considered just as important as those of traditional scientists. The aim of this paper is to present the software implementation process of DiaFit, alongside the app itself.

## Methods

### Agile Software Development

For the purposes of this paper, the terms *client* and *stakeholder* are used interchangeably, and refer to physicians and citizen scientists. To ensure that a given software meets the needs of the client, development requires significant communication between the development team and the client [12]. When following a traditional waterfall software development lifecycle model [13], the initial phase of software production aims at producing a software requirement specification document [12]. This document describes the software simply, unambiguously, and entirely, from its architectural design to its simplest functionalities and behaviors. This approach is not well-suited for rapid software development [19]. Moreover, this approach significantly delays the presentation of functional prototypes to the stakeholders. Therefore, we followed best practices in software engineering by using an Agile software development methodology [19]. Using Agile, we followed adaptive planning and evolutionary development principles, aiming to deliver the software product early with continuous improvement.

We aimed to develop a fully integrative app that could be used to help patients manage their T2D and obesity, in a primary care setting and under the supervision of a primary care provider. We assembled a key stakeholder team comprised of citizen scientists (RB, CM, CRD), an internal medicine physician (ER), researchers in biomedical informatics, nutrition, and obesity science (FM, MC), and a software development team (TM, ZL). The citizen scientists were paid volunteers from the University of Florida Clinical and Translational Science Institute citizen scientist program. All citizen scientists had a chronic disease; either obesity and/or diabetes. The citizen scientists acted as de facto customers for the DiaFit software, whereas the clinician had both a customer and consultant role in the project.

We followed an Agile software development methodology [19], with an emphasis on the following principles of the Agile manifesto:

1. Continuous and regular delivery of software components to allow the users to provide feedback early in the process, and to engage the intended users.

2. A project involving highly motivated individuals.

3. Regular face-to-face meetings.

4. Frequent and close cooperation between the stakeholders and continuous refinement of the design.

5. Functional software as main metric for progress.

6. Simplicity (ie, make the software as simple as possible, in collaboration with the stakeholders).

7. Pair-programming, whereby two programmers work together on a part of the code, with one programmer writing the code (the driver) and the other programmer assessing for correctness (the observer).

### Requirements and Preference Elicitations

During the initial phase of the project we reviewed the limitations of existing apps (see *Comparison With Prior Work* in the *Discussion* section) [6,10,11]. We then identified the key elements necessary for the self-management of T2D and obesity, and how they could be addressed effectively using a mobile health (mHealth) approach. This phase led us to a first set of basic software requirements, resulting from best practices for T2D and obesity management, and the end-user software requirement elicitation process. The initial set of requirements generated is summarized in Table 1.

**Table 1:** Basic requirements.

Requirement	Functional Requirement
The patient-user should be able to use application on an iPhone	DiaFit runs on the following devices running iOS 9.2 or newer: iPhone 5 to iPhone 6s Plus
The patient-user should have secure access to their account	DiaFit requires username and password for access. Passwords are saved via the keyed-hashed message authentication code - secure hash algorithm 1 random *salted* for each password, and standard encryption protocol
The patient-user should be able to track their eating habits	DiaFit provides user with access to a large nutrition database for logging dietary intake
The patient-user should be able to measure calorie intake	DiaFit calculates the calorie intake of the user, utilizing the food consumption that is input by the user, and the nutritional database
The patient-user should be able to measure carbohydrates, proteins and fats	DiaFit provides a graphical breakdown of the macronutrients consumed by the user, based on food consumption that is input by the user, and the database information
The patient-user should be able to measure calorie expenditure	DiaFit supports Fitbit devices, Apple watch, or the iPhone on which the DiaFit app is installed, and provides the following calorie expenditure: (1) energy requirement estimate [20], calculated using the data that is input by the user; and (2) physical activity energy expenditure estimate, calculated utilizing the information gathered by the device selected by the user
The patient-user should be able to track blood glucose	DiaFit allows the user to track their glucose by inputting their current glucose value, utilizing any type of glucometer
The patient-user should be able to keep track of their medication	DiaFit provides data entry for the user to manually enter any medication and, if desired, create a reminder based on the time that medicine is taken

**Figure 1:**
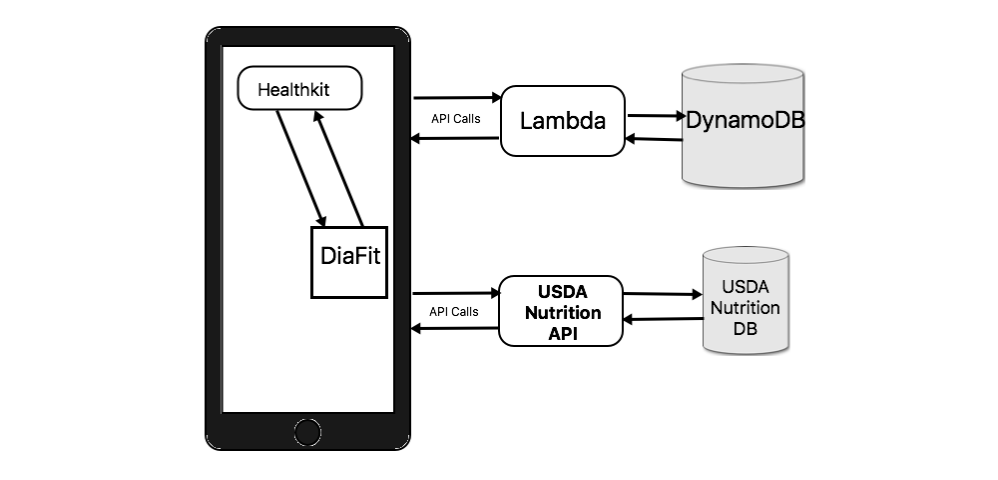
Architecture of DiaFit. API: Application Program Interface; DB: database; USDA: United States Department of Agriculture.

DiaFit was implemented using iOS, so the software engineering team chose Apple’s Swift 2 programming language. This approach allowed for a tight integration with the current (and future) functionalities of Apple Healthkit (a platform for collecting data from various health and fitness apps in iOS). We used a serverless back-end developed with Amazon Web Services (AWS) Lambda and AWS DynamoDB database. The use of AWS Lambda allowed the app to have high availability and scalability without provisioning or managing servers. AWS also provided Health Insurance Portability and Accountability Act compliance, as well as Family Education Rights and Privacy Act and Federal Information Security Management Act compliance, if needed. We used the United States Department of Agriculture National Nutrient Database Application Program Interface (API) [21] to allow the users to search for their food consumed, and allow the app to calculate and save the nutritional information. The architecture of DiaFit is described in Figure 1.

Additionally, we are in the process of incorporating the RxNorm API [22] into DiaFit. This addition will allow the user to access the RxNorm medication database in order to facilitate and increase the accuracy of medication entry, versus the current option of free text input. DiaFit will be made available through GitHub, under the GNU General Public License agreement, version 3 [23]. By making DiaFit open source, the diabetes community at large will have the opportunity to contribute to, refine, and expand the functionalities of the tool, promoting cooperation and innovation. Following the Agile software development methodology, the software team started the implementation of DiaFit with partial requirements, which was generated after the initial preference elicitation meeting with the physician and citizen scientists. Using Agile allowed us to present incomplete, albeit functional, versions of DiaFit to the stakeholders of DiaFit. Therefore, an effort was made to ensure modularity of the various components of the app.

Additional requirements were identified in subsequent meetings, referred to as *sprints* in the Agile methodology, with the stakeholders of the DiaFit project. We incorporated a validated measure of well-being (Diabetes-Specific Patient-Reported Outcome Quality of Life [24]) and patient-physician communication functionality, through direct secure messaging in DiaFit. Patient-reported outcomes (PROs) are important validated measures that can be used to measure the quality of life of patients [25], and therefore provide a method to track changes. However, a tradeoff needs to be made between obtaining data through phone prompts and interfering with patients’ lives. PROs can only be collected via direct manual entry of the data in the app, so we worked with citizen scientists to assess how often PROs should be elicited. Consensus was reached on PROs being entered by patients in DiaFit 1.0 when they chose to, rather than have an app prompt requesting the data from the user. Additional focus groups in a primary care setting allowed us to answer this question more accurately. Moreover, the team acknowledged that an essential functionality of DiaFit should be the ability to seamlessly upload data pertaining to physical activity and glucose to DiaFit, via Bluetooth and BLE. These requirements are summarized in Table 2.

**Figure 2:**
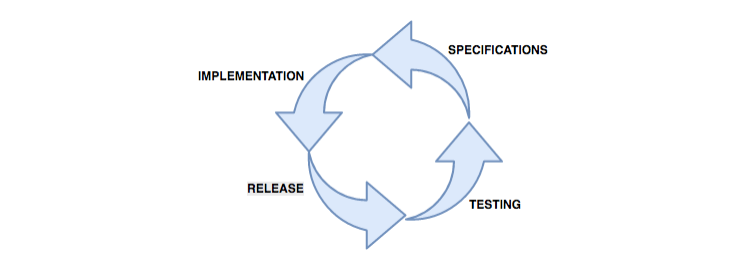
Agile methodology for DiaFit development.

Conceptually, the interactive process between the research team, software team, and the *clients* is summarized in Figure 2. The specifications were obtained through a continuous and highly interactive process led by the development team, primarily via face-to-face weekly meetings with the research team, and from face-to-face monthly meetings with the physician and citizen scientists. Intermediate DiaFit versions were first presented to the research team for evaluation and feedback. Release was then presented to the *clients,* then tested for usability and functionality.

**Table 2:** Additional partial requirements.

Requirement	Functional Requirement
The patient-user should be able to monitor their well-being	DiaFit prompts the user weekly for Short Form (9 questions) Diabetes-Specific Patient-Reported Outcomes Quality of Life (National Institutes of Health [NIH] Patient-Reported Outcomes Measurement Information Systems [PROMIS]) [24], and stores PRO responses
The patient-user should be able to track physical activity with a variety of wearables	DiaFit allows user to synchronize with iPhone Apple step counter, iWatch, and FitBit devices, and stores steps in a database
The patient-user should be able to track blood glucose	DiaFit allows the user to track their glucose by automatically detecting if glucose data has been saved to Apple Health by a Bluetooth glucometer (ie, the wireless smart glucose-monitoring system from iHealth)
The patient-user should be able to receive feedback from their physicians	DiaFit allows patient-user to give access data logs to physician-user
The physician-user should be able to see summary statistics of their patients	DiaFit provides physician view
The physician-user should be able to send encouragement messages to patient-user	DiaFit provides secure messaging interface
The patient-user should be able to read physician-user messages	DiaFit provides secure access to messages

## Results

### Dimensions

DiaFit incorporates account information and the following dimensions: nutrition, physical activity, blood glucose, medication, and Diabetes-Specific Patient-Reported Outcomes Quality of Life [24], as well as measures of subjective [26] and objective socioeconomic status information for research purposes. For privacy reasons, data entry is entirely voluntary, and participants may choose to leave fields blank. DiaFit supports Bluetooth data uploads for physical activity and glucose monitoring.

### Icon, Login, and Account Information

**Figure 3:**
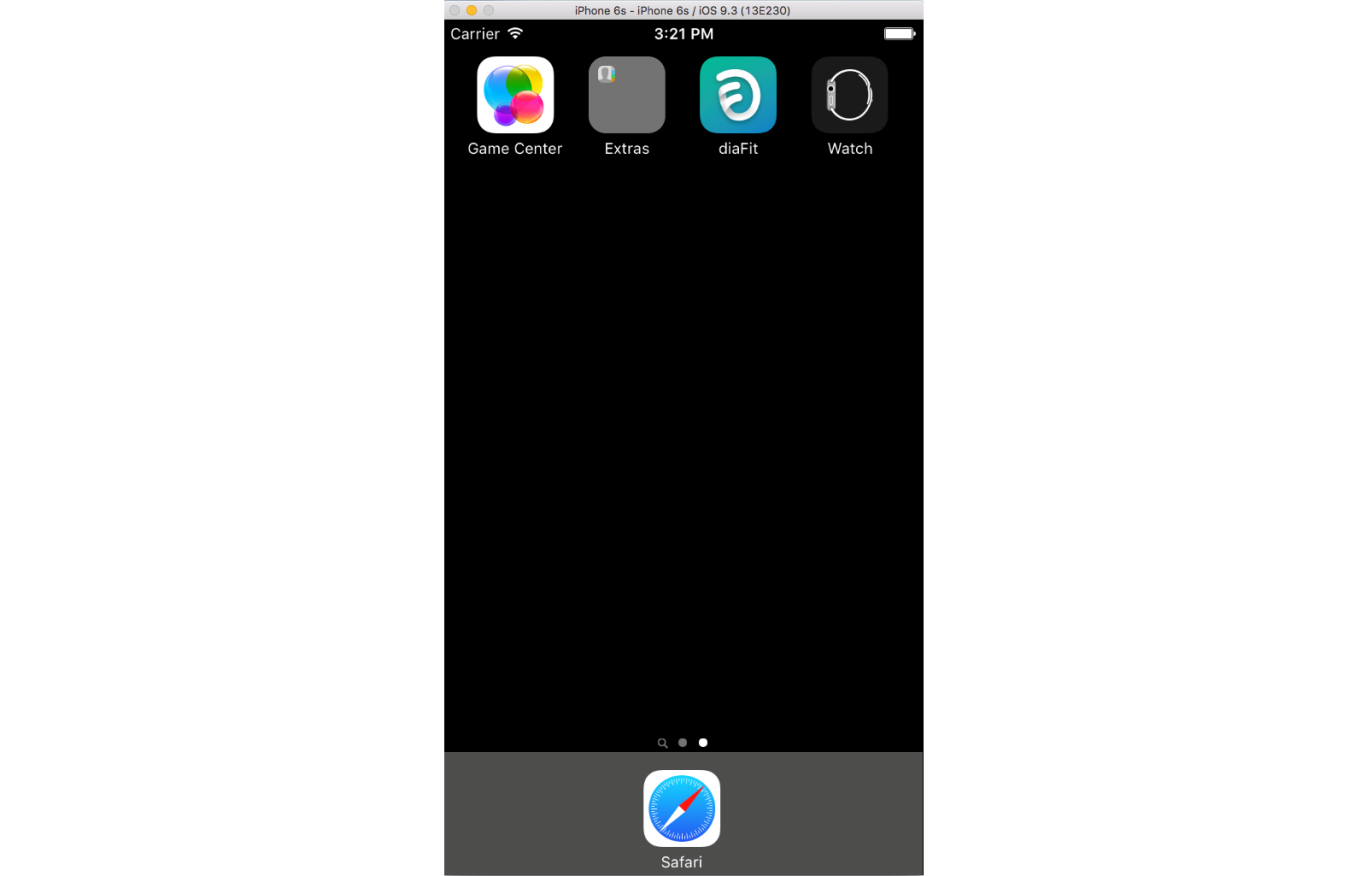
DiaFit icon.

**Figure 4:**
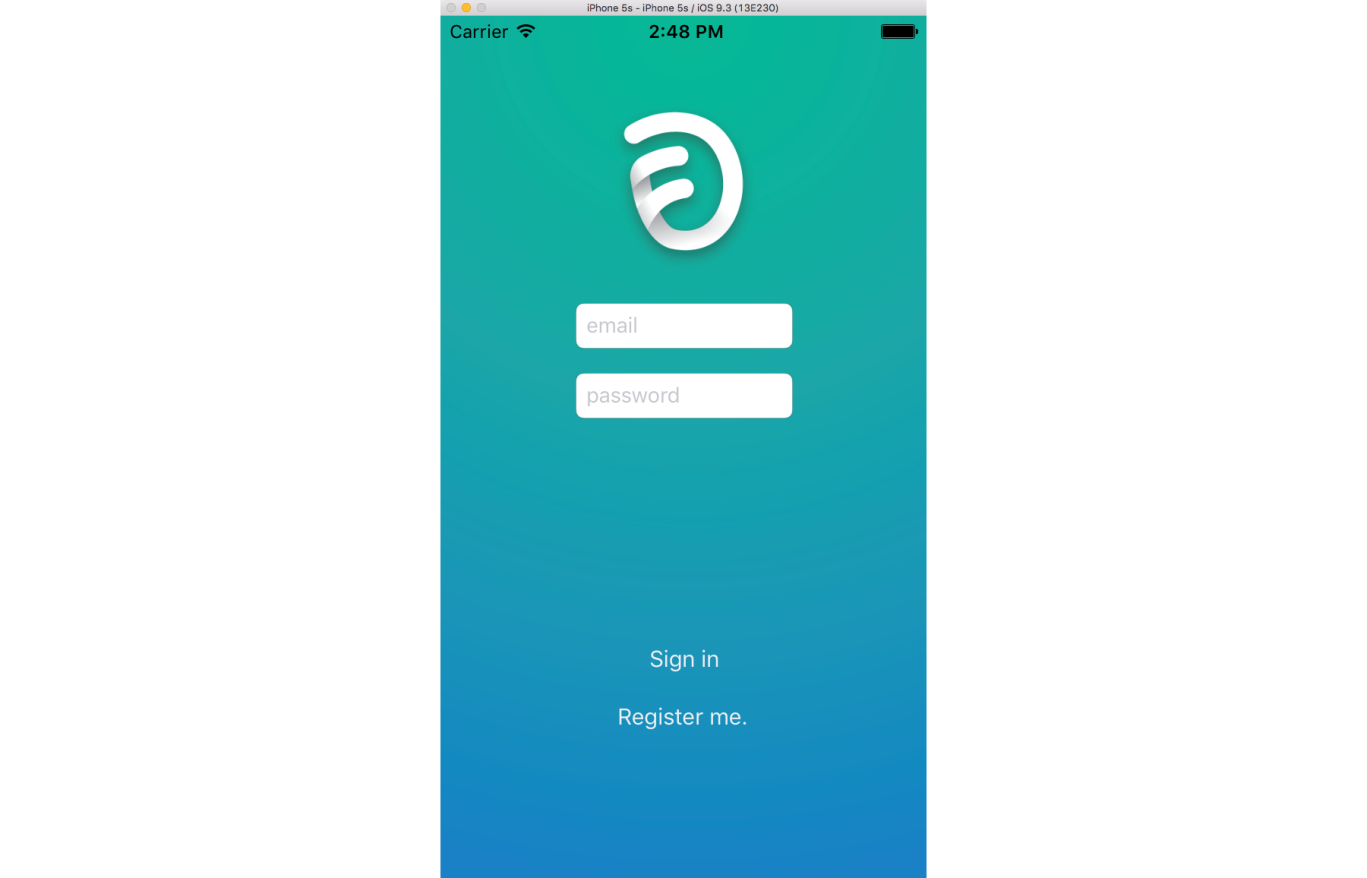
Login/sign-in.

**Figure 5:**
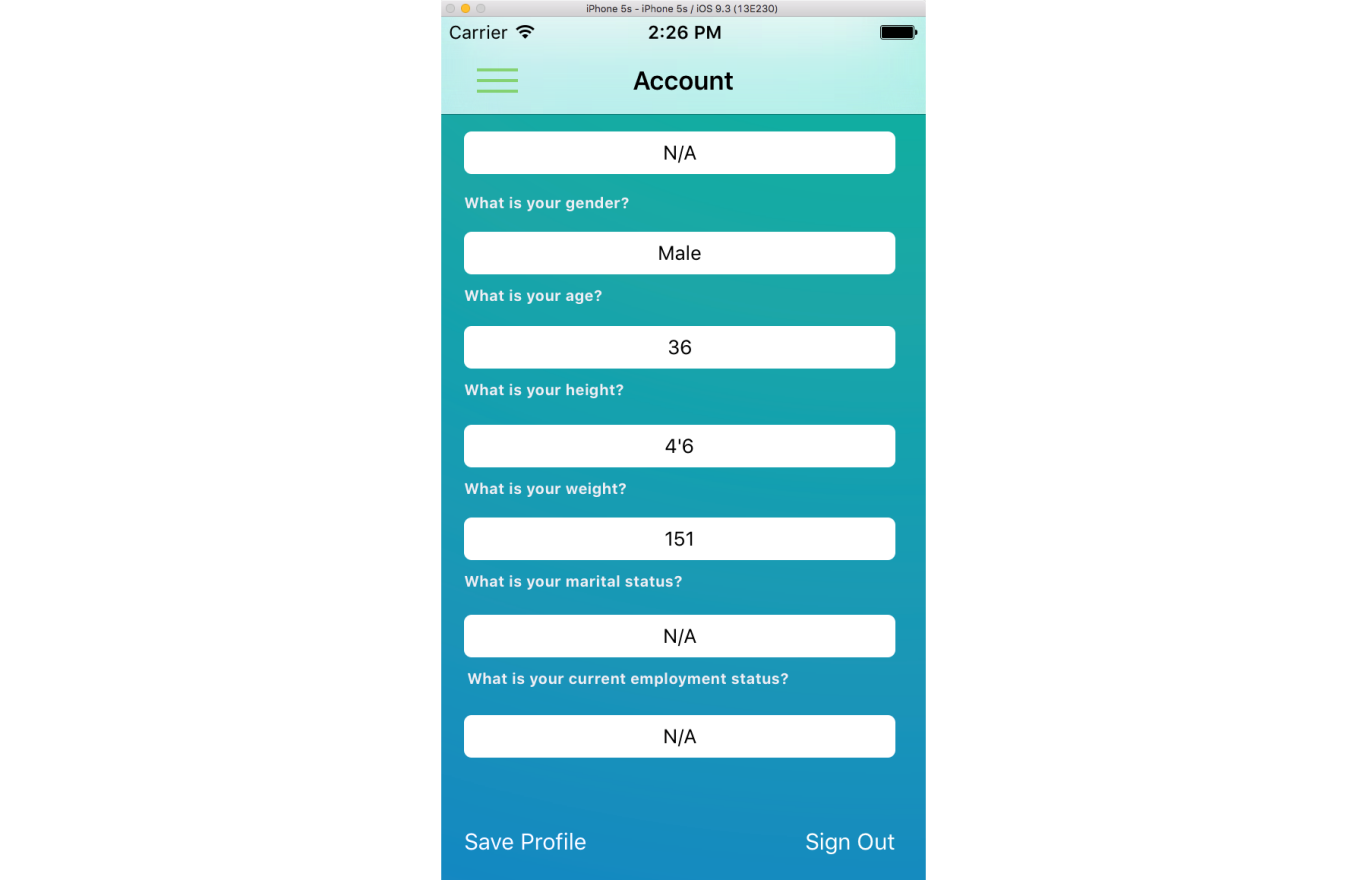
Account information.

DiaFit’s icon was developed with the larger group of citizen scientists (Figure 3). The objectives were to have a meaningful icon for the intended users, as well as an icon that is found quickly on a phone, to increase the likelihood of app use, and thus adherence. The login screen is pictured in Figure 4. The basic demographics that we included in our account information screen (Figure 5) include gender, age, height, weight, marital status, and employment status.

### Key Functionalities

**Figure 6:**
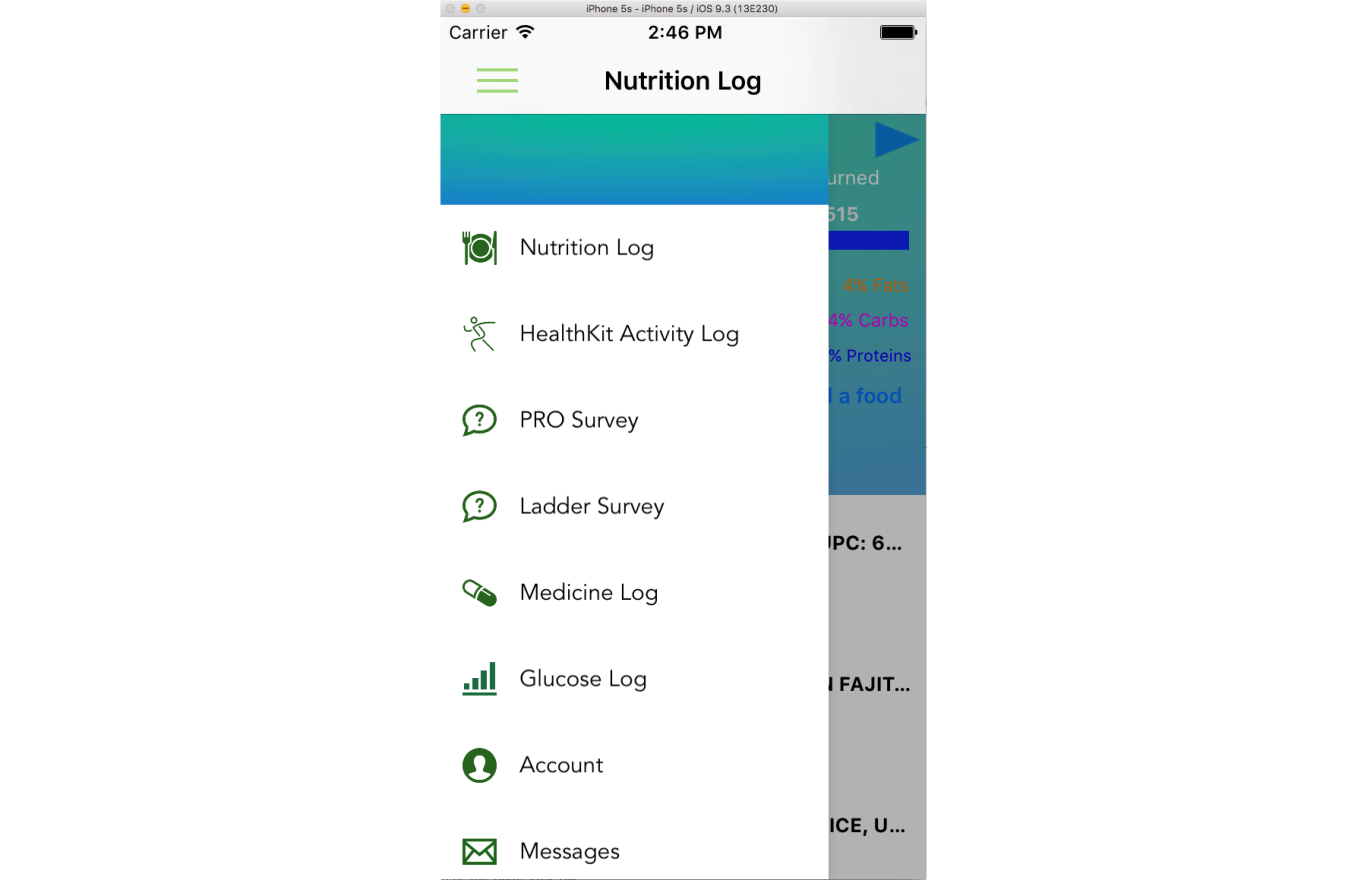
Slider menu.

**Figure 7:**
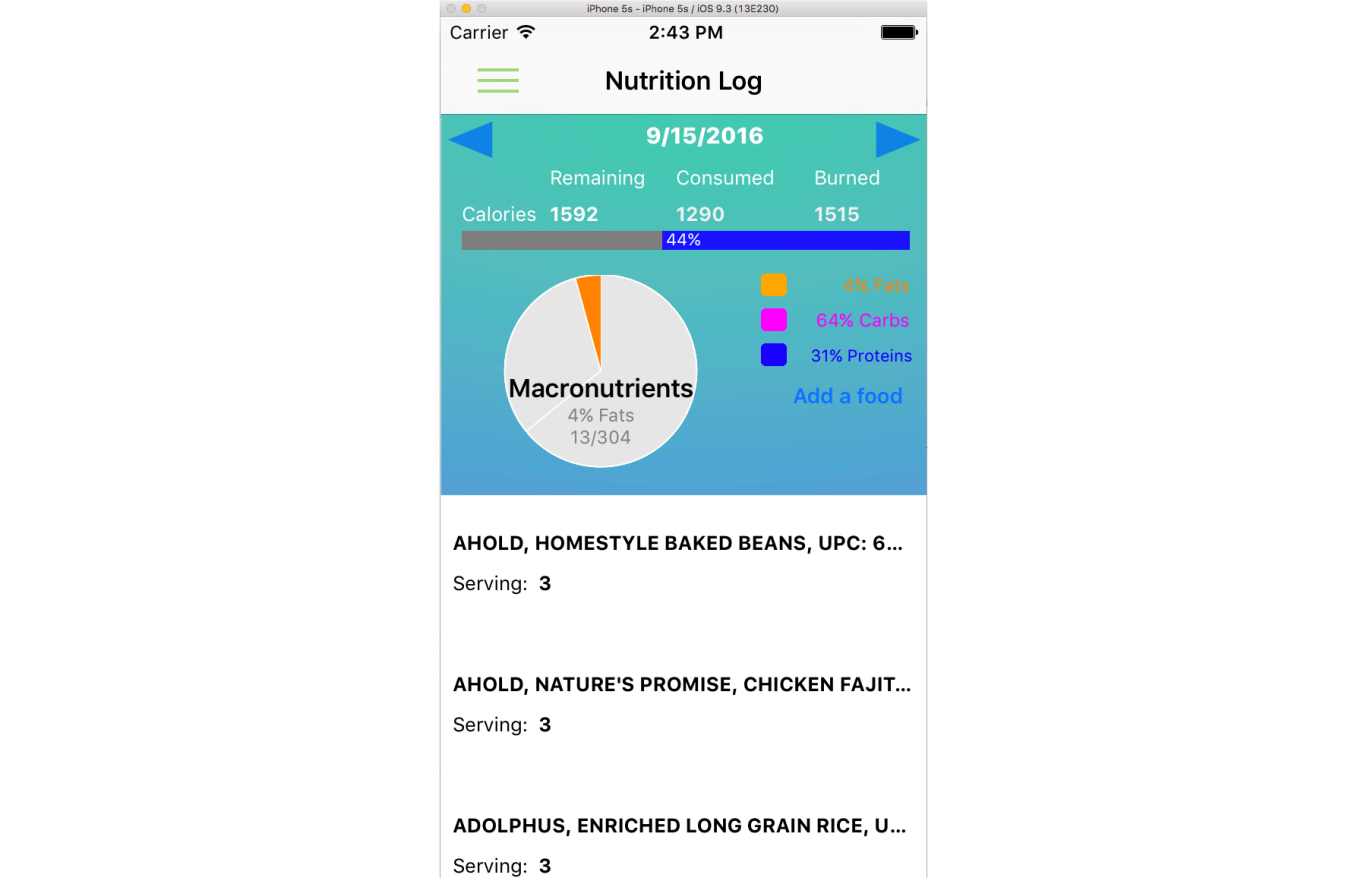
Nutrition tracking.

**Figure 8:**
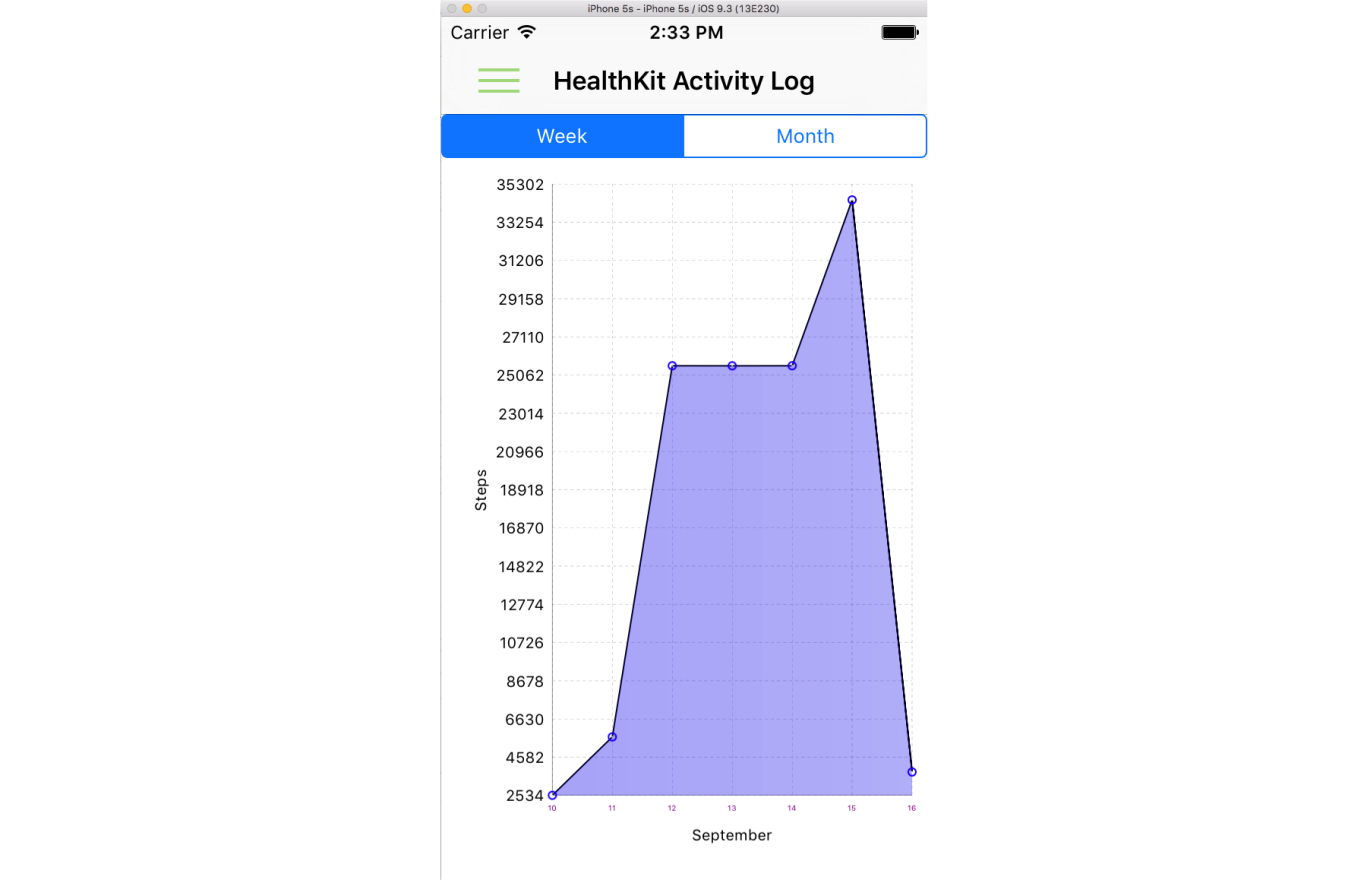
Physical activity log.

**Figure 9:**
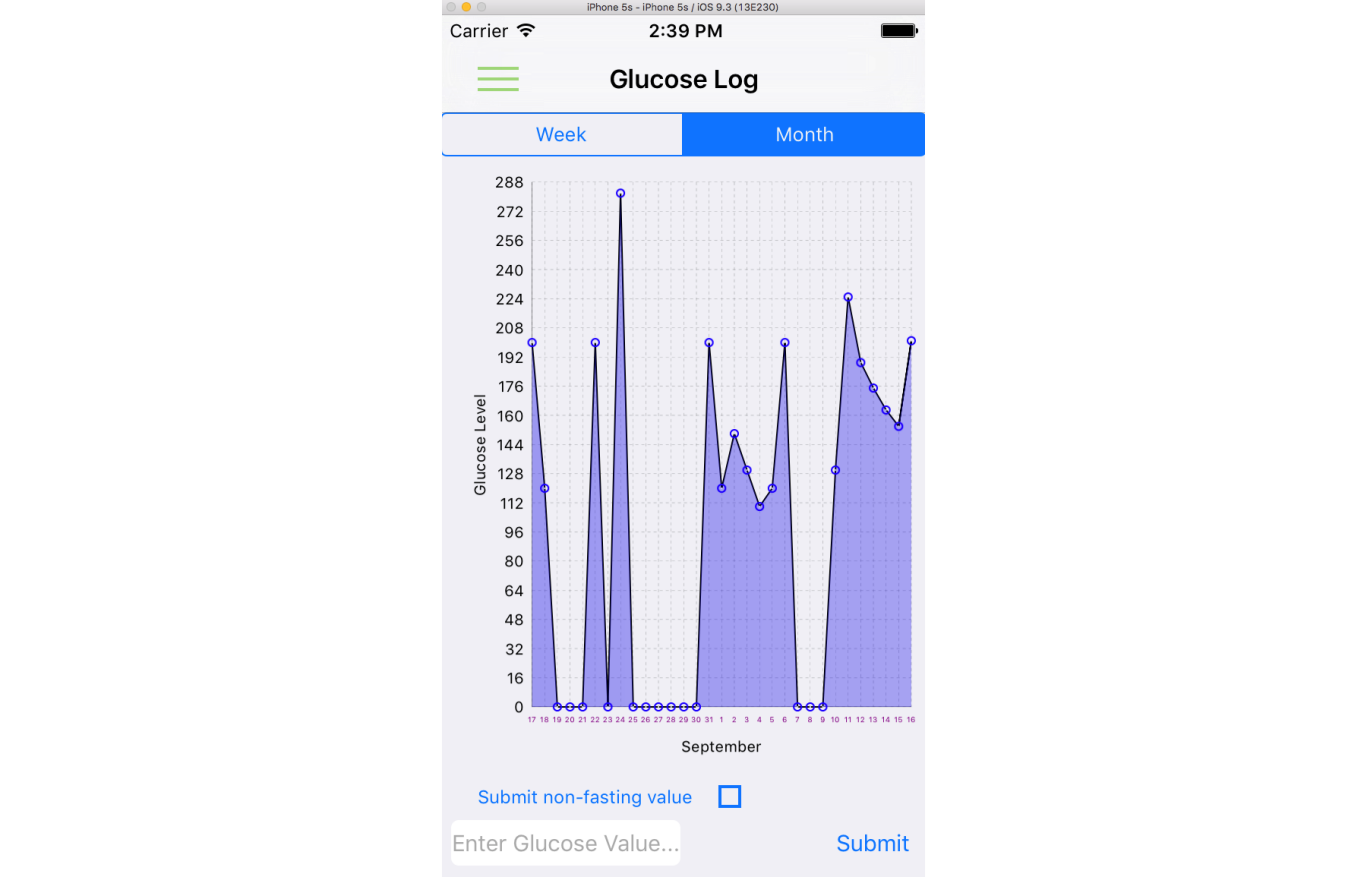
Glucose log.

**Figure 10:**
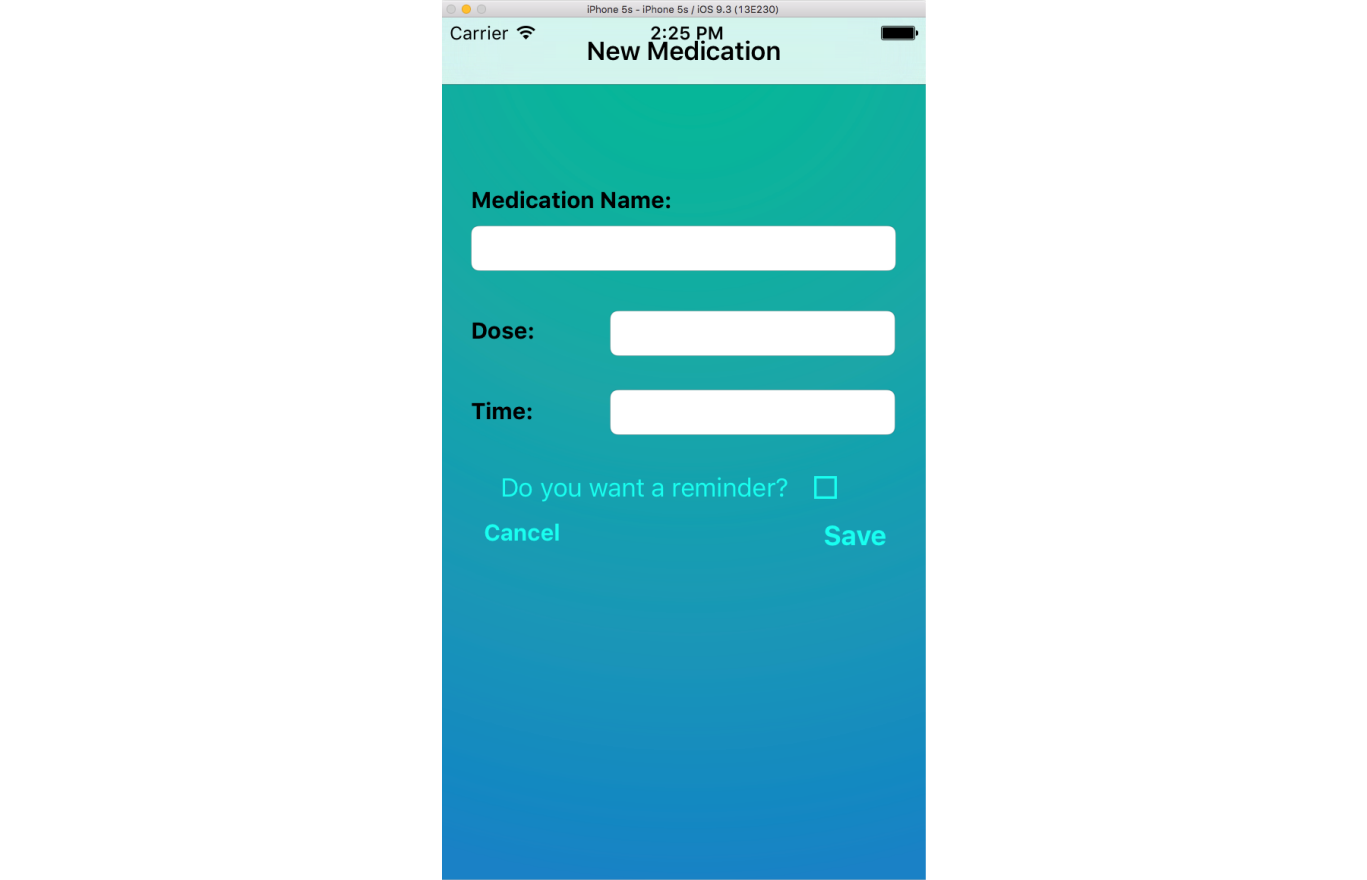
New medication entry.

The key functionalities of DiaFit were the following:

1. Access to a large nutrition database, which includes food items, calories, and breakdown in macronutrients, sodium, and fiber (Figures 6 and 7). Although micronutrients are an important aspect of a healthy diet, our discussions with the citizen scientists suggested that this would likely lead to information overload, and may not be not critical to our target end-users.

2. Physical activity tracking and seamless data entry. For the first version of the DiaFit app, the software team focused on integration with iPhone activity data, Apple watch, and Fitbit devices (Figure 8).

3. Glucose monitoring, either through manual input or Bluetooth seamless upload with iHealth glucose monitors. Another feature of glucose entry is the possibility to differentiate fasting glucose versus nonfasting glucose, which can be specified by the user (Figure 9).

4. Medication use via manual data entry, although DiaFit is being improved with an RxNorm API (Figure 10).

5. PROs (Figure 11), using an NIH PROMIS short form quality of life assessment tool. These functionalities are described in Figures 4-11.

6. Simplicity was also identified as a key, albeit nonfunctional, requirement of DiaFit. Indeed, with an aging population with T2D and obesity, it is critical to make the app as simple as possible [10], which led our design choices. We opted for a slider menu (Figure 6) to allow for easy navigation through the various components of DiaFit, and we also opted for limited nutritional variables (beyond macronutrients) versus other popular nutrition/physical apps such as myfitnesspal.

Additionally, simple graphic capabilities were added to allow the user to track changes and see improvement over time. To continue our development process, our citizen scientists are undergoing software testing of the app and reporting bugs, frequency of bugs, and needed user interface changes through a Web-link.

### Physician View

Finally, DiaFit offers a physician view, which allows the monitoring of patient improvements remotely and asynchronously (Figure 12). The physician side of the app also offers basic secure messaging capabilities, allowing a physician to easily and securely send a text-based message to a patient. We are also working on adding machine learning-based automated messaging capabilities to DiaFit, which will be available in the next release of the app.

**Figure 11:**
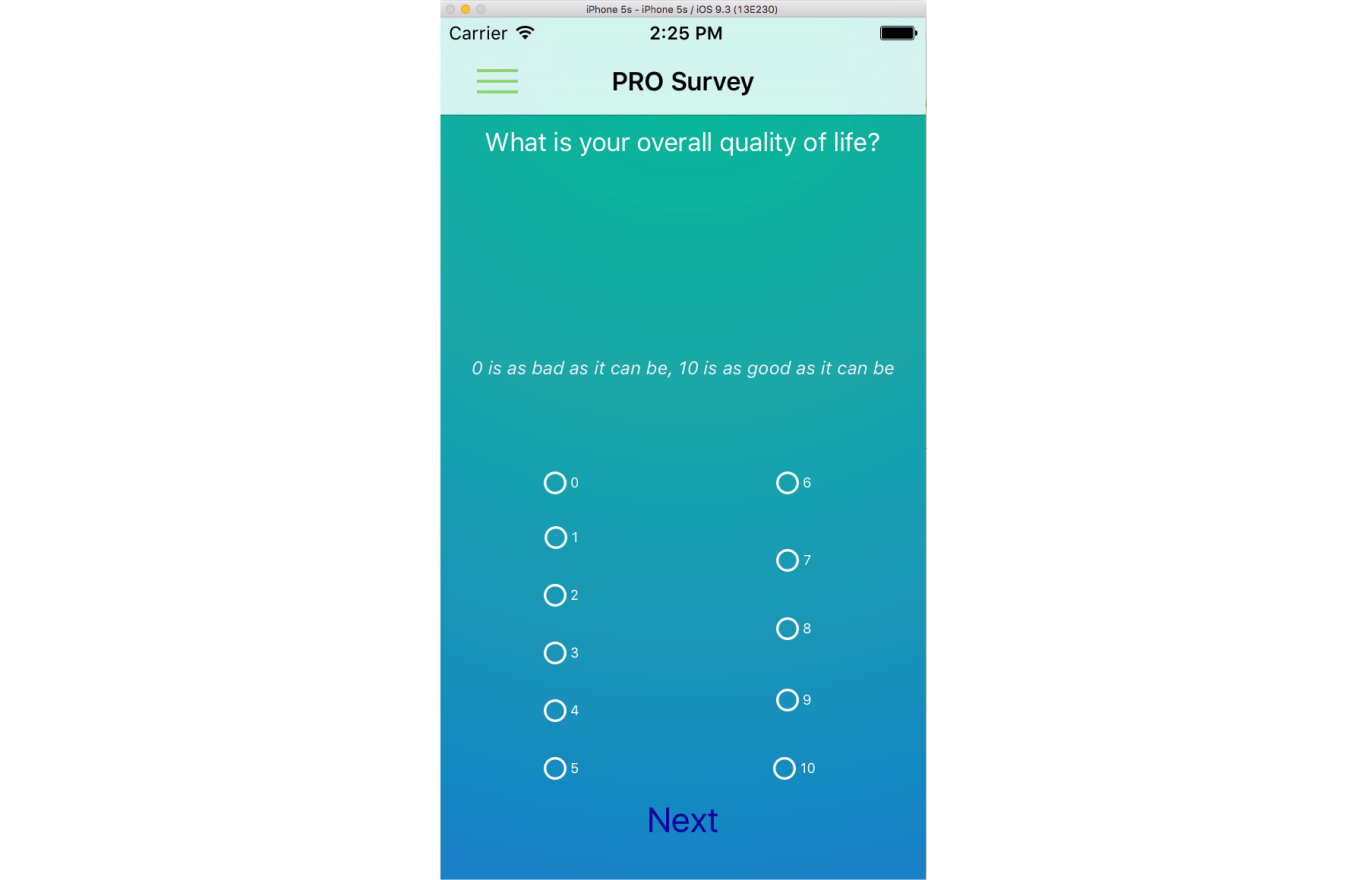
Patient-reported outcomes survey.

**Figure 12:**
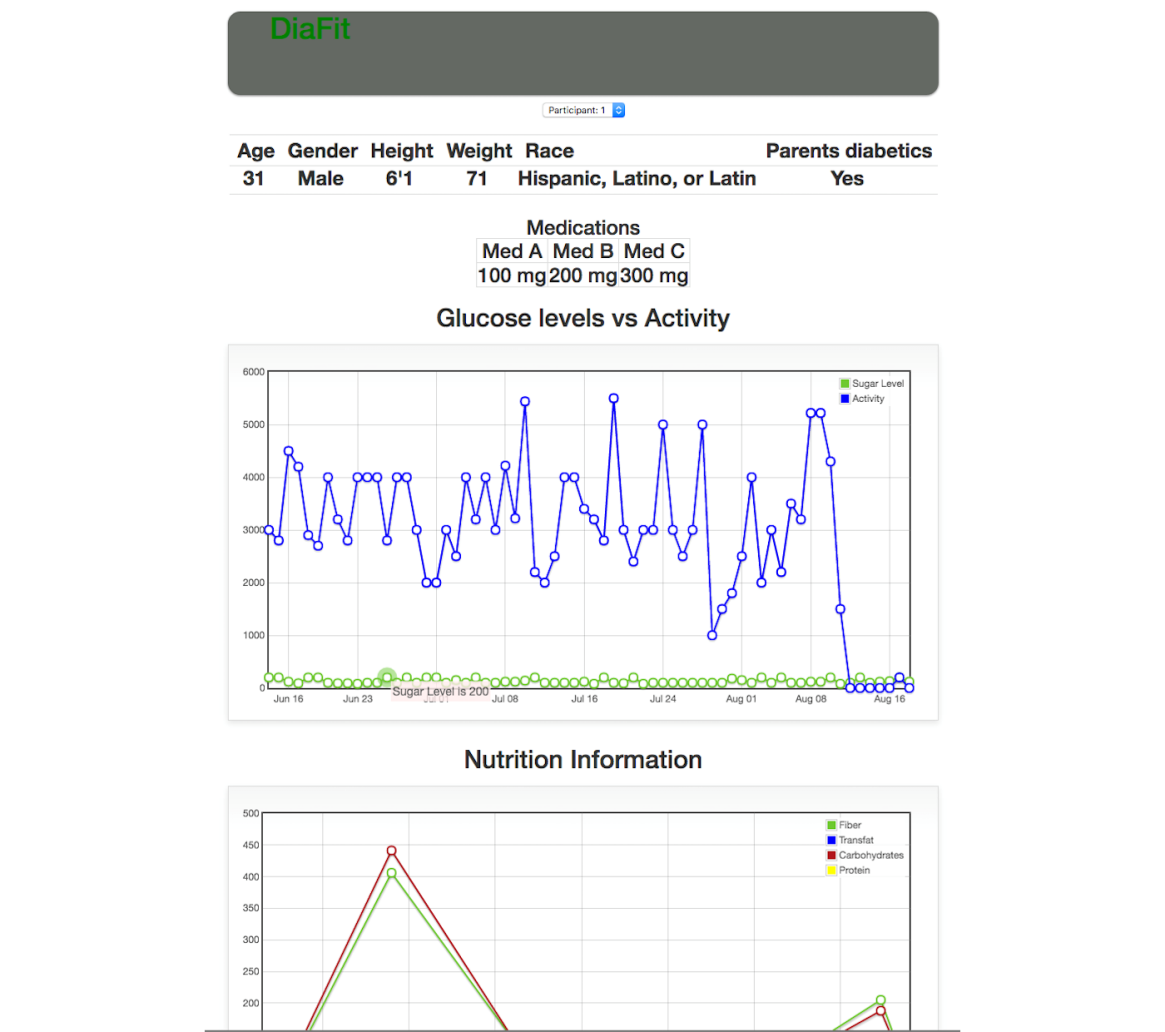
Physician view of DiaFit.

## Discussion

### Principal Results

The 2 main objectives of the DiaFit project were to (1) develop an evidence-based app that allows patients with T2D and obesity to manage their chronic conditions, and (2) ensure that the app is developed with the end-user in mind by involving them in the entire development of the product, rather than only in the testing phase (as is commonly done). To achieve this, we assembled a team of highly motivated individuals comprised of biomedical informaticians, nutrition and obesity science researchers, software engineers engaged in app development, a primary care physician, and citizen scientists with a strong interest in mHealth as a tool to address chronic conditions. To the best of our knowledge, this is the first attempt at assembling such a diverse team that included all stakeholders in the development of an app for the management of chronic conditions. It is important to note that our approach differed from that of focus groups, and that all team members acted as de facto collaborators on this project, bringing in a diverse range of expertise and perspectives. Although we included evidence-based components in the development of DiaFit, we cannot yet state that we have successfully created an evidence-based app because the effectiveness of the app has not yet been tested. Thus, whether we have succeeded in making the app evidence-based will need to be tested in future studies, and currently remains outside the scope of this paper.

The initial meeting with all DiaFit constituents occurred in late January 2016. The design and implementation phase started in late March 2016 due to the difficulty of recruiting motivated iOS developers. Finally, the deployment of the current version of DiaFit occurred mid-August 2016. The main barrier to accelerating the development process proved to be scheduling. Coordinating meetings with several citizen scientists who work full time, and a physician with long clinical hours, resulted in the research and development teams deciding to have partial team meetings to elicit feedback for improvement. However, the high motivation of all involved parties ensured that deadlines were met, and deliverables were presented on time to DiaFit stakeholders.

### Limitations and Lessons Learned

The development of DiaFit presented several challenges. Primary care physicians are significantly time-constrained. Therefore, careful planning is necessary to schedule Agile sprints early at the beginning of the project. We had not accounted for planning issues adequately at the beginning of this project, and subsequently lost a significant amount of time in early stages. Given the necessity for short time periods between meetings (ie, short Agile sprints), the development process should clearly lay out bimonthly meetings from the initial phase of the project, rather than letting development drive meeting times. However, our initial delays were mitigated by very strong clinical support for DiaFit. Having a clinician championing such a project is essential, not only to ensure sufficient feedback, but also to increase chances of adoption at later time points. Based on the expertise of our team, we decided to focus our efforts on iOS development and ignored the large Android market. With a growing segment of the population speaking Spanish, we also need to make the app available in Spanish. We are currently in version 1 of the app, and have not yet moved on to the staging that incorporates automated messaging, which would help the patients handle interactions related to diet, physical activity, and their glucose responses, which would be beneficial for self-management of T2D or obesity. Finally, DiaFit has not yet been tested as part of a pragmatic trial in a primary care setting with patients and physicians. However, prior work on mHealth strategies for diabetes management suggests that DiaFit could have a significant positive impact on patients’ lives [11]. Development of DiaFit for Android, a Spanish version of DiaFit, and assessment of the DiaFit in a primary care setting (internal medicine) are planned as part of our future work.

### Comparison with Prior Work

Most apps developed for managing T2D and obesity do not include all variables that need to be addressed for these chronic conditions. Such apps typically address one dimension only, such as glucose monitoring or nutrition tracking, and often omit key functionalities that facilitate data entry and adherence, such as Bluetooth compatibility [6,10,11]. Moreover, to the best of our knowledge, no diabetes-related app attempts to link nutrition, physical activity, glucose monitoring, and medication use with PROs, thus missing critical patient feedback for quality of life with a chronic condition. App creation also lacks patient and physician involvement [10], and therefore lacks essential feedback from the targeted users. Finally, very few apps on the market are available open source, despite several attempts at democratizing health data, such as the Open mHealth initiative [27].

### Conclusions

Despite the presence of >100,000 health and fitness-related apps in the Apple store alone, apps tend to be of poor quality with regards to clinical evidence. Very little effort has been placed in developing apps while including the potential end-users (eg, patients and physicians or health care professionals) in the process. In this paper, we presented the iterative process and design of the DiaFit process development, an app created to help patients with T2D and obesity manage their conditions more effectively. The process was based on the creation of a team representing all constituents of the DiaFit project, and we involved them as clients in an Agile software development project. We believe that this approach will allow academicians interested in mHealth strategies to close the gap between *fun* apps and evidence-based apps, and allow mHealth to reach its goal of revolutionizing health care by improving scalability of access. Finally, we hope that providing DiaFit as an open source solution to diabetes and obesity management will lead the community to improve and grow its functionalities to better serve patients.

## Abbreviations

API: application program interface
AWS: Amazon Web Services
BLE: Bluetooth Low Energy
mHealth: mobile health
NIH: National Institutes of Health
PRO: patient-reported outcome
PROMIS: Patient-Reported Outcomes Measurement Information Systems
T2D: type 2 diabetes
